# Therapeutic Targeting of Oxidative Phosphorylation in Microsatellite Instability-High Gastric Cancer

**DOI:** 10.7150/jca.127225

**Published:** 2026-03-30

**Authors:** Bing Ang, Yi Bai, Xiyue Deng, Qiong Wu, Yuqian Wang, Shanshan Xu, Weixin Zhang, Yang Li, Dapeng Chen, Ruixi Li, Siyang Li, Zhigang Zhao, Yamin Zhang

**Affiliations:** 1 Department of Medical Oncology, Tianjin First Central Hospital, School of Medicine, Nankai University, Tianjin, China.; 2 Department of Hepatobiliary Surgery, Tianjin First Central Hospital, School of Medicine, Nankai University, Tianjin, China.; 3 School of Medicine, Nankai University, Tianjin 300071, China.; 4 Tianjin First Central Hospital Clinic Institute, Tianjin Medical University, Tianjin 300192, China.

**Keywords:** gastric cancer, MSI, oxidative phosphorylation, PDX, ATP

## Abstract

**Background:**

Gastric cancer is the third leading cause of cancer-related mortality worldwide. According to The Cancer Genome Atlas (TCGA), it can be classified into four molecular subtypes, including microsatellite instability (MSI) and genomically stable (GS) subtypes, which display distinct clinical and pathological features. However, differences in their tumor microenvironment, particularly metabolic reprogramming, remain poorly understood.

**Methods:**

Single-cell RNA sequencing data from gastric cancer patients classified as GS or MSI were enrolled. Cell clusters were identified and annotated to compare cellular landscapes between subtypes. Differential gene expression and pathway analyses were performed among malignant epithelial cells. Key genes related to oxidative phosphorylation were identified using LASSO regression, and their expression was further validated in the TCGA dataset. Patient-derived xenograft models were used to compare tumor growth rates, ATP levels, and expression of oxidative phosphorylation -related genes.

**Results:**

Single-cell transcriptomic analysis revealed eight major cell types in MSI tumors. Compared to the GS subtype, MSI samples showed significantly greater infiltration of T cells and a lower proportion of epithelial cells. Malignant cells from MSI samples exhibited increased activity of oxidative phosphorylation pathways. LASSO regression identified five oxidative phosphorylation-related genes that were consistently overexpressed in MSI tumors in both single-cell and TCGA datasets. In Patient-derived xenograft models, MSI tumors grew more rapidly and demonstrated higher ATP levels and elevated expression of the five oxidative phosphorylation-related genes compared to MSS tumors.

**Conclusion:**

Our study reveals enhanced oxidative phosphorylation metabolism in MSI gastric cancer at single-cell resolution and identifies five oxidative phosphorylation-related genes that may serve as potential therapeutic targets for this subtype.

## 1. Introduction

Gastric cancer is one of the most common malignant tumors worldwide and poses a major threat to public health^1^. Recent advances in molecular biology have revealed that gastric cancer is not a single disease, but rather a collection of molecularly distinct subtypes, each with unique clinicopathological features. In 2014, The Cancer Genome Atlas (TCGA) classified gastric cancer into four molecular subtypes based on genomic profiling: microsatellite unstable (MSI), chromosomally unstable (CIN), Epstein-Barr virus (EBV) positive, and genomically stable (GS)^2^. Similarly, the Asian Cancer Research Group (ACRG) identified MSI as one of four molecular subtypes with distinct characteristics and clinical outcomes^3^. The high heterogeneity of gastric cancer has limited the efficacy of conventional treatments, making molecular subtyping essential for advancing personalized therapy.

MSI subtype gastric cancer accounts for 15-20% of cases^4^ and is characterized by deficient DNA mismatch repair (dMMR), leading to microsatellite instability^5^, ^6^. Microsatellites are short, repetitive DNA sequences prone to replication errors^7^. Inactivation of MMR genes—through genetic or epigenetic alterations—results in dMMR, which confers a high tumor mutation burden (TMB) and genomic instability^8^, ^9^. The MSI-H/dMMR phenotype is associated with high immunogenicity and robust immune infiltration, making it susceptible to immune checkpoint inhibitors^10^. Although some patients with MSI gastric cancer exhibit sensitive and durable responses to immunotherapy, primary resistance remains a challenge^11^-^14^. Potential mechanisms are as follows: tumor cells may evade immune attacks by downregulating antigen presentation pathways (e.g., MHC molecules) or secreting immunosuppressive factors (e.g., TGF-β)^15^; chronically activated T cells may lose effector functions, leading to reduced cytokine production and impaired proliferation^16^. Beyond intrinsic immunogenicity, tumor cell-intrinsic metabolic states and microenvironmental metabolic regulation may also play critical roles in determining tumor progression and therapeutic responses. Emerging evidence suggests that a subset of MSI gastric cancer cells undergo metabolic reprogramming during treatment, thereby enhancing therapy resistance or facilitating immune evasion.

In recent years, with the advancement of molecular classification of gastric cancer, increasing attention has been directed toward the roles of metabolic pathways across distinct molecular subtypes. Among these, the mitochondria-dependent oxidative phosphorylation (OXPHOS) pathway, a central axis of cellular energy production, remains insufficiently characterized in gastric cancer, particularly in the MSI-H subtype. Accumulating evidence indicates that upregulation of OXPHOS promotes tumor cell tolerance to oxidative stress^17^, supports cancer stem cell-like properties^18^, and modulates immune effector function through metabolic competition and interactions with immune cells^18^. Collectively, these findings suggest that OXPHOS may be closely associated with the immune microenvironment, tumor progression, and therapeutic responsiveness in gastric cancer.

In this study, we used single-cell RNA sequencing (scRNA-seq) and patient-derived xenograft (PDX) models to investigate the tumor microenvironment and molecular pathways in MSI gastric cancer, with the goal of identifying potential therapeutic targets.

## 2. Methods

### 2.1 Tissue specimens

This study was approved by the Ethics Committee of Tianjin First Central Hospital. All patients enrolled in PDX experiments provided written informed consent.

### 2.2 scRNA-seq data processing and clustering

We obtained raw data from the GEO database (GSE183904). Based on tumor origin and molecular subtype, we included 7 MSI and 9 GS samples for subsequent analysis. Data processing was performed using Seurat (v5). We loaded the gene expression matrices from each sample and merged them into a single Seurat object. Cells were subjected to quality control with the following criteria: 1) removal of cells with mitochondrial, ribosomal, and hemoglobin gene percentages exceeding 20%, 40%, and 1%, respectively; 2) exclusion of cells where the number of unique feature counts (nFeature_RNA) was <200 or >5000, or the total UMI counts (nCount_RNA) was <400 or >35,000; 3) retention of genes detected in at least three cells. We predicted and removed potential doublets using DoubletFinder (v2.0.3). We corrected technical batch effects across samples using Harmony (v1.0). The integrated data were scaled and subjected to principal component analysis (PCA). We performed dimensionality reduction and visualization using Uniform Manifold Approximation and Projection (UMAP). Cell communities were constructed based on a shared nearest neighbor (SNN) graph in the Harmony-corrected space, and unsupervised clustering was performed using the Louvain algorithm at a resolution of 0.8 to define final cell subpopulations. We annotated cell clusters based on the expression patterns of canonical cell type-specific marker genes. The annotated cell type identities were stored as metadata (celltype) in the Seurat object for all subsequent analyses.

### 2.3 Evaluation CNVs from scRNA-seq data

We performed CNV evaluation using the inferCNV package (https://github.com/broadinstitute/inferCNV). We calculated CNVs of epithelial cells using the average gene expression from immune cells (B cells) as a reference. Default parameters were applied (cutoff = 0.1). The CNV score was calculated as the mean of the CNV regions.

### 2.4 Differential Gene Expression and Pathway Enrichment Analysis

To identify transcriptomic differences in epithelial cells between MSI and GS groups, we performed differential gene expression analysis. We used the FindMarkers function from the Seurat package to compare epithelial cells from MSI and GS cohorts. Genes with |log₂FC| ≥ 0.25 and p-value < 0.05 were classified as significantly upregulated, downregulated, or stable.

Kyoto Encyclopedia of Genes and Genomes (KEGG) pathway enrichment analysis was subsequently conducted on the up- and down-regulated gene sets separately using the enrichKEGG function from the clusterProfiler package.

### 2.5 Feature genes selection by LASSO

Epithelial cell subsets were isolated from annotated scRNA-seq data, and normalized gene expression matrices were obtained. To enhance model robustness and computational efficiency, low-abundance genes detected in fewer than 50% of cells were filtered out. Feature selection was subsequently performed using least absolute shrinkage and selection operator (LASSO) regression. The optimal regularization parameter λ was determined via 10-fold cross-validation, with the λ-1se value selected to promote model sparsity and generalizability. A discriminative gene signature distinguishing MSI from GS samples was identified based on genes with non-zero coefficients in the final model.

### 2.6 Validation of Gene Expression Using Public Databases

To validate the expression patterns of the identified signature genes, we analyzed RNA expression data from the Stomach Adenocarcinoma (TCGA, PanCancer Atlas) cohort available through the cBioPortal for Cancer Genomics (https://www.cbioportal.org/). Samples were categorized based on microsatellite instability (MSI) status, with MSI-high (MSI-H) defined as samples exhibiting more than 4 repeated microsatellite loci and microsatellite stable (MSS) samples defined as those with 4 or fewer repeats. Differential expression analysis of the characteristic genes was performed between these molecular subgroups to confirm their association with MSI status.

### 2.7 Xenograft nude mice model

For PDX experiments, tumors were grouped according to microsatellite status (MSI vs MSS), rather than TCGA molecular subtypes. NOD-SCID mice at 5 weeks were purchased (Beijing Vital River Laboratory Animal Technology Co., Ltd.) and housed under specific-pathogen-free conditions. Approximately 1 × 10^7^ tumor cells from MSI/MSS patients in 100 μl of PBS were implanted subcutaneously into the right armpits of nude mice. On the 14^th^ day, the mice were euthanized, and tumors were excised and photographed. Tumor weight and volume were recorded, with tumor volume calculated using the formula: Tumor volume = (length × width^2^)/2.

### 2.8 ATP investigation

We assessed ATP concentration in tumor tissue using an Enhanced ATP Assay Kit (Beyotime, Shanghai, China) according to the manufacturer's instructions.

### 2.9 Quantitative real-time polymerase chain reaction (qRT-PCR)

Total RNA was isolated from tumor tissues using TRIzol reagent (Vazyme Biotech, Nanjing, China). Complementary DNA (cDNA) was synthesized using the ExScript RT-PCR kit (TaKaRa, Japan) according to the manufacturer's protocol. Quantitative real-time PCR (qRT-PCR) was performed using SYBR Green Master Mix (Vazyme Biotech) in quadruplicate reactions. β-actin served as the endogenous control for normalization. Relative gene expression was quantified using the 2-ΔΔCt method. The primer sequences were listed in Table 1.

### 2.10 Statistics

Statistical analysis of all data was performed using R (v4.0.2) and Python (v3.79) software. Student's t-tests were conducted for normalized distributed data, Mann-Whitney test was used for abnormal distributed data. All figures are marked by distinctive symbols indicating statistical significance (ns: P > 0.05; *: P ≤ 0.05; **: P ≤ 0.01; ***: P ≤ 0.001; ****: P ≤ 0.0001).

## 3. Results

### 3.1 Profile of gastric scRNA-seq data

**Figure 1:**
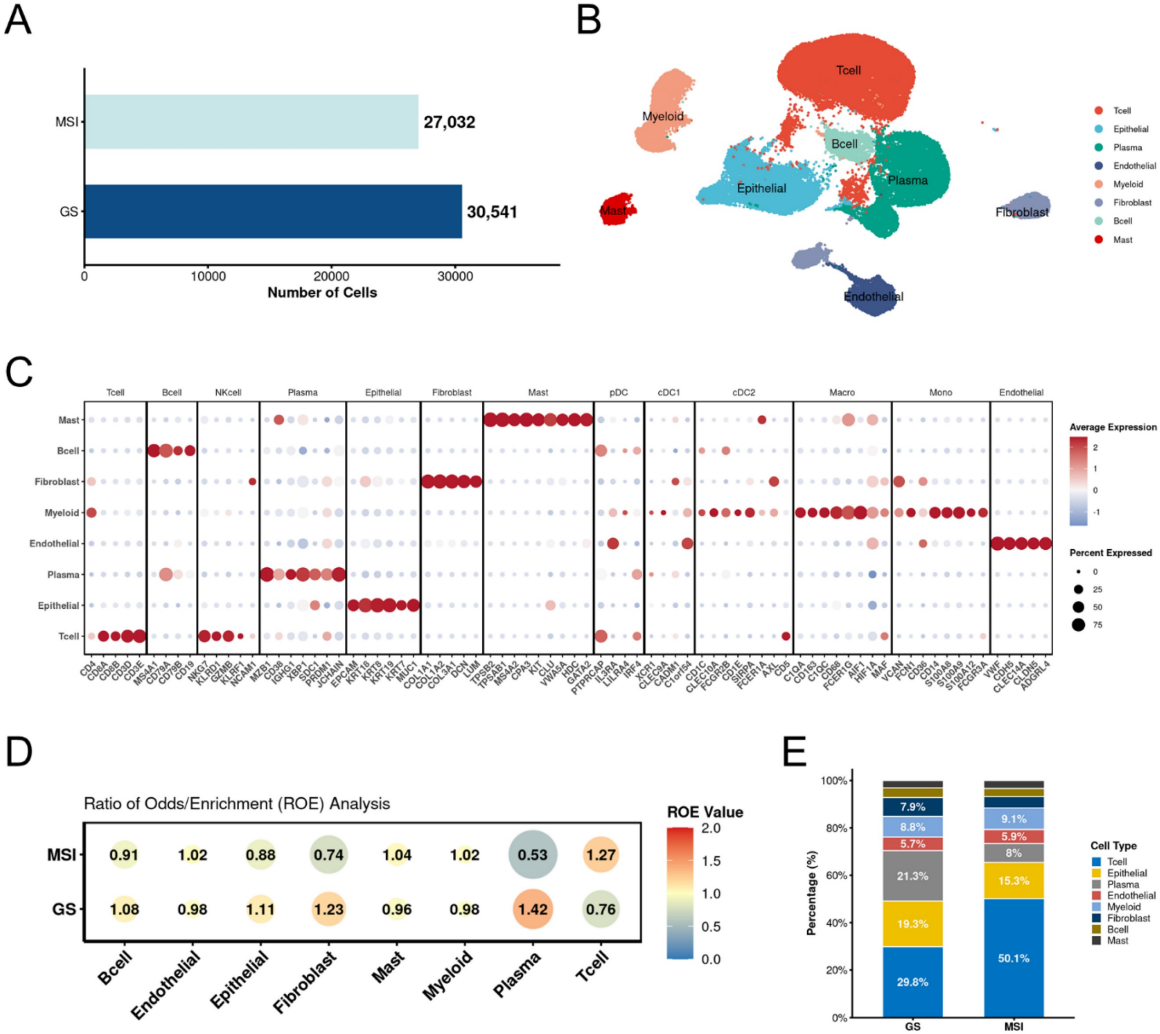
** Overall result of gastric cancer scRNA-seq data. A.** Bar plot shows the cell numbers of each group. **B.** The annotated major cell types. Each type was labeled with special color. **C.** Dot plot of canonical marker genes for major cell types. **D.** Major cell type estimated by R_O/E_ between MSI and GS groups. **E.** Bar plot of percentage for major cell types between MSI and GS groups. MSI: microsatellite instability; GS: genomically stable.

We first generated the RNA expression matrix for each sample. After filtering out low-quality cells, we obtained a total of 57,573 cells, including 27,032 cells from the microsatellite instability (MSI) group and 30,541 cells from the genomically stable (GS) group (Figure 1A). Based on these 57,573 cells, we performed clustering and manually annotated them into eight subtypes according to the expression of canonical marker genes: B cells (MS4A1, CD79A, CD79B, CD19), plasma cells (MZB1, CD38, IGHG1, XBP1, SDC1, PRDM1, JCHAIN), T cells (CD4, CD8A, CD8B, CD3D, CD3E), endothelial cells (VWF, CDH5, CLEC14A, CLDN5, ADGRL4), epithelial cells (EPCAM, KRT18, KRT8, KRT19, KRT7, MUC1), fibroblasts (COL1A1, COL1A2, COL3A1, DCN, LUM), mast cells (TPSB2, TPSAB1, MS4A2, CPA3, KIT, CLU, VWA5A, HDC, GATA2), and myeloid cells (C1QA, CD163, C1QC, CD68, CD36, CD14) (Figure 1B). Each cell type exhibited high expression of its respective canonical marker genes (Figure 1C). To further explore differences in cell type distribution between the two groups, we performed an analysis of the ratio of observed to randomly expected cell numbers (R_O/E_). The results demonstrated enrichment of T cells and depletion of epithelial cells in the MSI group, which is consistent with the known immunogenic nature of MSI tumors reported in previous studies (Figure 1D). Analysis of the proportional distribution of different cell types between MSI and GS groups further supported this observation (Figure 1E).

### 3.2 Oxidative phosphorylation enriched in MSI malignant tumor cells

**Figure 2:**
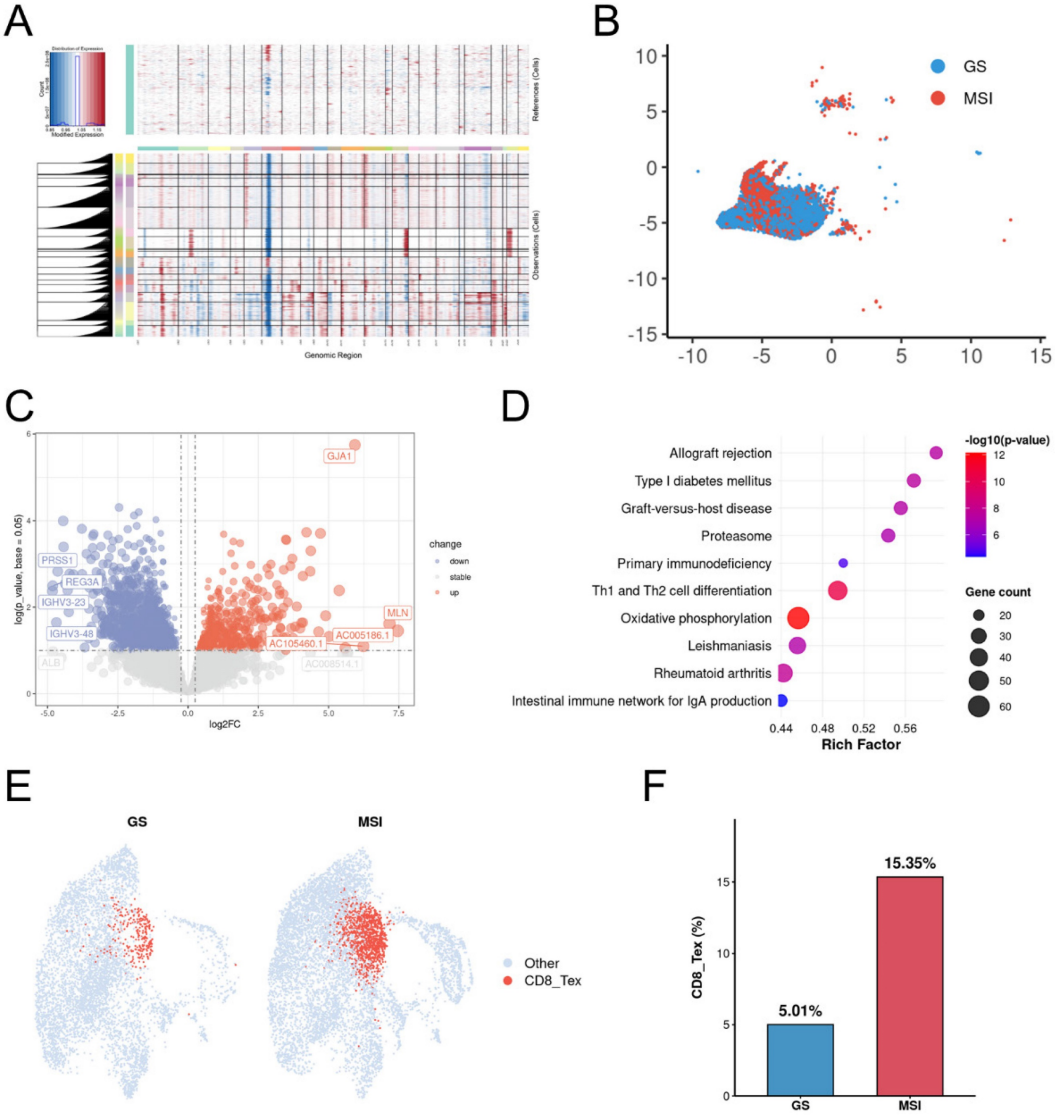
** Oxidative phosphorylation enriched in MSI Malignant tumor cells. A.** Heatmap showing large-scale CNV profile of epithelial cell for each group. Red and blue colors represent high and low CNV level, respectively. B cells are defined as reference cells. **B.** Distribution of epithelial cells in MSI and GS groups. **C.** Volcano plot of the differential expressed genes of epithelial cells between MSI and GS groups. **D.** The bar plot displays the top 10 KEGG pathways enriched for up-regulated genes in the MSI group. **E.** Dot plots of scores for CD8_tex cells between MSI and GS groups. **F.** Box plots of percentage for CD8_tex cells between MSI and GS groups. CD8_tex: Terminal exhausted CD8; MSI: microsatellite instability; GS: genome stable.

To investigate tumor cell dynamics, we isolated epithelial cells and performed large-scale chromosomal copy number variation (CNV) analysis using transcriptomic data with B cells as a diploid reference. Widespread chromosomal alterations were observed in epithelial cells from both MSI and GS samples, indicating pervasive malignant features (Figure 2A). Furthermore, malignant cells exhibited distinct clustering patterns between MSI and GS groups (Figure 2B). Differential gene expression analysis identified 3,868 upregulated and 2,806 downregulated genes in MSI compared to GS samples (Figure 2C). KEGG enrichment analysis revealed significant involvement of the oxidative phosphorylation pathway among differentially expressed genes (Figure 2D). Further analysis for CD8 T cells revealed the exhausted CD8 +T cells significantly enriched in MSI group (Figure 2E, 2F). These results indicated oxidative phosphorylation pathway may contribute to development of MSI gastric cancers.

### 3.3 Feature genes associating with MSI

**Figure 3:**
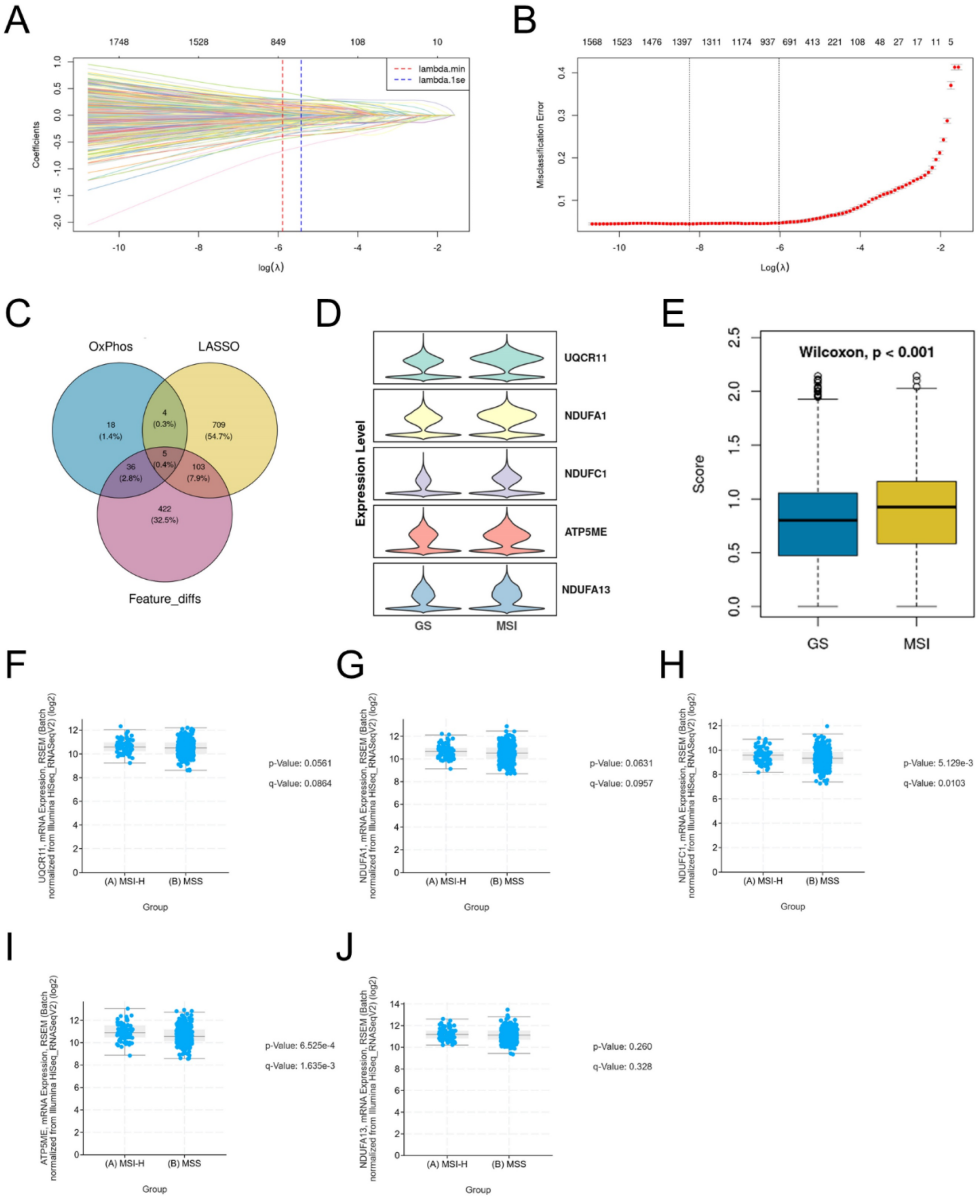
** Feature genes associating with MSI. A-B.** Lasso regression analysis of feature genes between MSI and GS groups. **C.** Venn diagram showing the overlap among LASSO-selected genes, oxidative phosphorylation-related genes, and differentially expressed genes detected in ≥50% of cells. **D.** Violin plots show the expression of the 5 overlapping genes in MSI and GS groups. **E.** Overall score of identified 5 genes between MSI and GS groups. **F-J.** Box plots show the expression of 5 genes in validation TCGA gastric cancer dataset.

To identify key gene features distinguishing MSI from GS epithelial cells, we performed feature selection using LASSO regression. We determined the optimal regularization parameter (λ.1se) through 10-fold cross-validation, resulting in a final model retaining 821 genes with non-zero coefficients (Table S1), which formed a compact and highly discriminative gene set. The coefficient distribution (Figure 3A) and partial likelihood deviation (Figure 3B) of the selected features are shown. We then intersected three gene sets: genes with non-zero coefficients from the LASSO model, differentially expressed genes detected in at least 50% of cells, and genes associated with oxidative phosphorylation. This integrative analysis identified five consensus candidate genes (Figure 3C). All five genes exhibited higher expression in the MSI group (Figure 3D), and the overall score also supported their elevated expression in MSI (Figure 3E). Furthermore, analysis of the Stomach Adenocarcinoma (TCGA, PanCancer Atlas) cohort revealed that these five genes were significantly upregulated in the MSI-H group compared to the MSS group (Figure 3F-J). These results indicate that these five genes influence the oxidative phosphorylation pathway in MSI gastric cancers.

### 3.4 Tumor growth and oxidative phosphorylation levels in PDX mice

**Figure 4:**
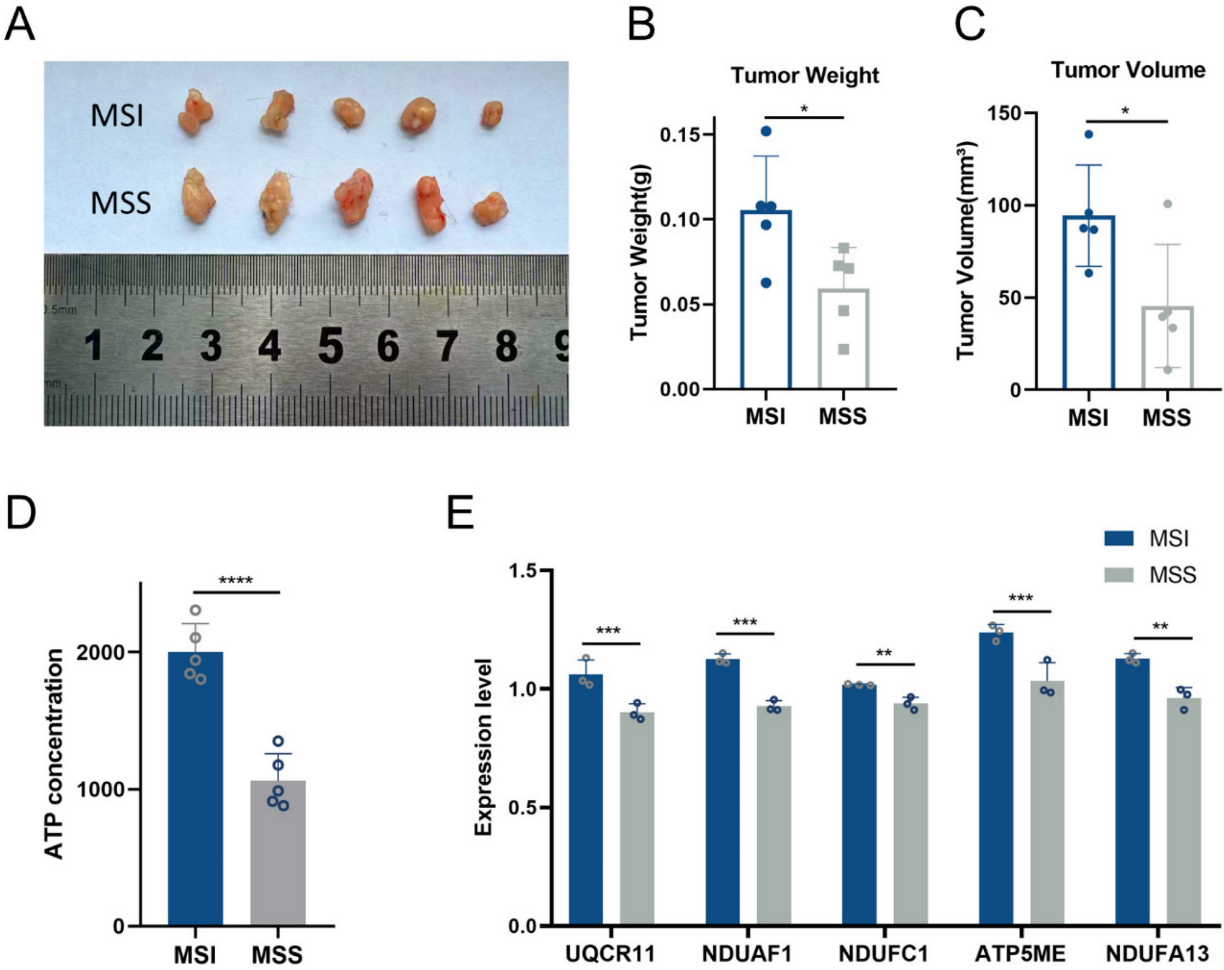
** Tumor growth and oxidative phosphorylation levels in PDX mice. A.**The Images of subcutaneous tumors in MSI (N=5) and MSS PDX mice (N=5) at 14^th^ day. **B.** Tumor weight of MSI (N=5) and MSS PDX mice (N=5) at 14^th^ day. **C.** Tumor volume of MSI (N=5) and MSS PDX mice (N=5) at 14^th^ day. **D.**ATP concentration of tumor tissue between MSI (N=5) and MSS PDX mice (N=5) at 14^th^ day. **E.** Core genes validation between MSI (N=5) and MSS PDX mice (N=5) at 14^th^ day. *: P≤0.05; **: P≤0.01; ***: P≤0.001.

To assess the impact of MSI and MSS classification on tumor growth, we subcutaneously implanted cells derived from MSI or MSS patients into mice and maintained them under identical conditions. On day 14, tumors in the MSI group exhibited significantly greater weight and volume compared to those in the MSS group (Figure 4A-C). We next evaluated oxidative phosphorylation levels in tumor tissues by measuring ATP production. The MSI group showed markedly elevated ATP levels relative to the MSS group (Figure 4D). Consistent with this, expression levels of five core genes involved in the oxidative phosphorylation pathway were also significantly upregulated in MSI tumors (Figure 4E). These findings collectively underscore the contribution of enhanced oxidative phosphorylation to the progression of MSI gastric cancers.

## 4. Discussion

Tumors with high microsatellite instability (MSI-H) are characterized by a high somatic mutation burden and increased frequency of insertions/deletions (INDELs) in microsatellite regions. When occurring in coding regions, these INDELs can generate tumor-specific neoantigens^19^. These neoantigens are recognized by T cells, triggering a robust T-cell response and promoting T-cell infiltration into the tumor bed. This abundance of antigenic targets, combined with an inflammatory tumor microenvironment (TME), leads to more effective anti-tumor immunity, which translates into a strong response to immune checkpoint blockade (ICB)^20^, ^21^.

Approximately 15%-30% of sporadic gastric cancers (GC) are MSI-H. These MSI-H GCs are more common in elderly female patients and are generally associated with lower rates of lymph node metastasis, earlier pTNM stage, and better prognosis^22^-^24^. These improved outcomes may be attributed to activated anti-tumor immunity reducing metastatic potential, along with high expression of immune checkpoint molecules such as PD-L1^25^. However, even among MSI-H GCs, responses to checkpoint inhibition and clinical outcomes vary, likely due to heterogeneity in the tumor immune microenvironment (TIME). Chronic exposure to neoantigens has been proposed to lead to T-cell exhaustion^26^ and infiltration of regulatory T cells^27^, fostering an immunosuppressive TME that undermines early immune surveillance. In this study, scRNA-seq analysis revealed that the transcriptional landscape of MSI gastric cancer reflects a coexistence of high immunogenicity and adaptive immune resistance—specifically, simultaneous enrichment of T cells and exhaustion of CD8⁺ T cells within the TME. This suggests that the emergence of immunosuppressive mechanisms may be a critical factor in MSI tumor progression.

Beyond the well-established immune context, our study further reveals significant upregulation of the oxidative phosphorylation (OXPHOS) pathway in MSI-H GC. This highlights a previously underappreciated driver of MMR-deficient tumor progression: metabolic reprogramming of malignant cells toward OXPHOS. OXPHOS, the primary metabolic pathway in mitochondria, generates ATP via the electron transport chain. Recent studies have linked OXPHOS to remodeling of the tumor immune microenvironment and therapy resistance^28^, ^29^. Certain MSI GCs exhibit transcriptomic signatures of stronger immunosuppression and T-cell exhaustion, which correlate with poor prognosis^30^.

Under therapeutic pressure, MSI gastric cancer cells may upregulate OXPHOS activity to facilitate adaptation and survival. Enhanced OXPHOS supports tumor growth under hypoxic or nutrient-competitive conditions^31^. Tumor cells relying on OXPHOS release metabolic byproducts such as lactate and reactive oxygen species (ROS), which can impair the function of T cells and NK cells^32^. Tumors with high OXPHOS activity may alter the metabolic state of the TME, reducing the efficacy of immunotherapy^33^. During immunotherapy, OXPHOS-upregulated tumor cells exhibit enhanced metabolic adaptability, potentially evading immune attack through bolstered antioxidant defenses and reduced antigen presentation^34^. Moreover, crosstalk between OXPHOS and other metabolic pathways, such as glycolysis, complicates therapeutic strategies^35^. Thus, Integrating our single-cell findings with existing evidence, we reasonably speculate that upregulation of OXPHOS may represent a novel, non-genetic mechanism by which MSI tumor cells reshape the immune microenvironment. Specifically, enhanced OXPHOS activity may influence immune cells through metabolic competition and altered redox signaling, thereby impairing CD8⁺ T cell effector function and promoting T cell exhaustion, ultimately contributing to tumor progression and therapeutic resistance.

Subsequently, we identified five OXPHOS-related signature genes in MSI gastric cancer. These genes do not function independently but instead represent key components of the mitochondrial electron transport chain or the ATP synthesis machinery. ATP5ME encodes a subunit of mitochondrial ATP synthase and is directly involved in ATP production. Dysregulation of ATP synthase components has been associated with aggressive tumor behavior and metabolic adaptability across multiple cancer types^36^. NDUFA1, NDUFA13, and NDUFC1 are essential subunits of mitochondrial complex I, which serves as the entry point of the electron transport chain. Complex I activity is not only critical for efficient ATP production but also plays a central role in maintaining redox homeostasis and regulating mitochondrial reactive oxygen species (ROS) levels^37^. Previous studies have shown that complex I dysfunction or adaptive upregulation is associated with cancer cell survival under oxidative and therapeutic stress, suggesting that enhanced expression of these subunits may contribute to the metabolic reprogramming observed in MSI gastric cancer cells. UQCR11 is a component of mitochondrial complex III and is required for electron transfer from ubiquinol to cytochrome c, thereby playing a crucial role in maintaining mitochondrial respiration. Alterations in complex III components have been shown to influence tumor progression through modulation of mitochondrial ROS signaling and energy production^38^. The concurrent upregulation of these five genes indicates a coordinated enhancement of mitochondrial oxidative phosphorylation. By supporting efficient ATP production and preserving mitochondrial function, elevated OXPHOS activity may enable tumor cells to survive under conditions of nutrient limitation, hypoxia, or therapeutic stress. In the context of MSI gastric cancer, characterized by a high mutational burden and associated cellular stress, such metabolic reprogramming may play a particularly important role.

Notably, in immunodeficient PDX models, MSI tumors exhibited faster growth compared to microsatellite-stable (MSS) tumors. This indicates that, in the absence of immune control, MSI tumor cells-driven by efficient OXPHOS metabolism-possess stronger intrinsic proliferative capacity. Therefore, the clinical behavior of MSI gastric cancer can be viewed as a dynamic balance between intrinsic tumor growth drive and external immune control. We hypothesize that ICB-insensitive MSI-H cases with poorer prognosis may be associated with OXPHOS metabolic dominance, which drives rapid proliferation and promotes immunosuppression.

Based on these mechanisms, targeting the OXPHOS pathway may represent a powerful synergistic strategy. Combining immune checkpoint inhibitors with OXPHOS-targeting agents could simultaneously release the brakes on the immune system and cut off the energy supply of tumors, offering a new direction to overcome treatment resistance and improve patient outcomes. The five-gene OXPHOS signature identified in our study may serve as a potential biomarker for selecting patients who could benefit from such metabolism-immunity combination therapies.

## 5. Summary

In this study, we systematically characterized the tumor microenvironment and metabolic features of molecular subtypes of gastric cancer by integrating scRNA-seq with PDX models. At the single-cell level, our analyses revealed distinct cellular compositions and metabolic activities between MSI and GS gastric cancers, and notably identified a marked activation of the OXPHOS pathway in MSI tumors. We subsequently defined a five-gene OXPHOS-related signature, which was validated across independent external datasets and *in vitro* experiments.

Using PDX models, we further demonstrated that MSI tumors exhibit increased ATP production and accelerated tumor growth, consistent with a tumor cell-intrinsic metabolic advantage associated with elevated OXPHOS activity.

Collectively, these findings highlight OXPHOS as a central metabolic feature of MSI gastric cancer progression and suggest a potential link between tumor-intrinsic metabolic states and immune dysfunction. Our study provides new insights into the metabolic heterogeneity of gastric cancer and supports the notion that targeting mitochondrial metabolism may represent a promising strategy to complement current therapeutic approaches for MSI gastric cancer.

## Supplementary Material

Supplementary table.

## Figures and Tables

**Table 1:** The primer sequences of qPCR.

primers	Primer sequence
ATP5ME-F	5'-CAGGTCTCTCCGCTCATCAAG-3'
ATP5ME-R	5'-GTCCAAAGAGTGGGTCGCAG-3'
NDUFA1-F	5'-TCATTGTTAAACACTCTGGGTTCG -3'
NDUFA1-R	5'-GGTCCACACTGCCCAGC-3'
NDUFA13-F	5'-TGGGCCCATCGACTACAAAC-3'
NDUFA13-R	5'-CGTACAGCTCCCCGATCAAG-3'
NDUFC1-F	5'-TTACCACGCTGGTGTAGTCTCA-3'
NDUFC1-R	5'-atgggggagttgaagggagaat-3'
UQCR11-F	5'-GCAGCCCACTGAAACTTACCA-3'
UQCR11-R	5'-TTGGAGAAAGGGTCCAGAGCA-3'
β-actin-F	5'-CATGTACGTTGCTATCCAGGC-3'
β-actin-R	5'-CTCCTTAATGTCACGCACGAT-3'

## Data Availability

The datasets involved in our work are available in the GEO (https://www.ncbi.nlm.nih.gov/geo/).

## Abbreviations

UMI): Asian Cancer Research Group (ACRG
